# Growth, Health, and Economic Performance of Post-Weaning Lambs Fed Alternative Concentrate

**DOI:** 10.3390/ani16081203

**Published:** 2026-04-15

**Authors:** Said Al-Khalasi, Abdullah Al-Ghafri, Fahad Al-Yahyaey, Suad Al-Saqri, Nasser Al-Habsi, Abdullahi Idris Muhammad

**Affiliations:** 1 UNESCO Chair on Aflaj Studies and Socio-Hydrology, University of Nizwa, P.O. Box 33, Nizwa 616, Oman; a.alghafri@unizwa.edu.om; 2 Animal Production Research Center, Ministry of Agricultural, Fisheries Wealth and Water Resources, P.O. Box 494, Muscat 320, Oman; fahad.al-yahyaei@mafwr.gov.om; 3 Department of Biological Sciences and Chemistry, University of Nizwa, P.O. Box 33, Nizwa 616, Oman; suad@unizwa.edu.om; 4 Department of Food Science and Nutrition, Sultan Qaboos University, P.O. Box 50, Muscat 123, Oman; habsin@squ.edu.om (N.A.-H.); abdullahimuhammad300@gmail.com (A.I.M.)

**Keywords:** feed efficiency, agricultural by-products, *Moringa oleifera*, economic viability, lamb nutrition

## Abstract

The adoption of cost-effective feeding regimes is essential to maintain the growth and physiological status of lambs after weaning. The economic benefits of an alternative concentrate diet for Omani lambs were also assessed. Twenty male Omani lambs (12.31 ± 3.22 kg; 5 months old) were allocated to a commercial concentrate (control) or formulated concentrate group (n = 10/treatment) for 63 days. The formulated concentrate consisted of date palm fronds (29%), barley (20%), fish meal (12%), date syrup (10%), *Moringa oleifera* (28%), and salt (1%), with all ingredient proportions expressed on a dry matter (DM) basis. Growth performance, feed efficiency, hematological indices, serum biochemical parameters, and cost analyses were evaluated using individual lambs as the experimental unit. Growth performance, feed efficiency, hematological and serum biochemical parameters, and cost analyses were performed. Final body weight (BW), total weight gain (TWG), average daily gain (ADG), and feed conversion ratio (FCR) were similar between the groups (*p* > 0.05). The average daily feed intake was similar between groups (*p* = 0.321). However, the feed cost per kg of gain and feeding cost were lower (*p* < 0.001) by 63 and 58%, respectively, for the formulated concentrate group. Time × group interactions were observed for red blood cell parameters, with higher platelet counts (*p* < 0.001) and reticulocyte hemoglobin content (*p* = 0.018) in the formulated concentrate group. The serum biochemical parameters changed over time, with similar responses between the groups. Appropriate substitution of ingredients in lamb concentrate diets with agricultural by-products positively influenced economic efficiency.

## Simple Summary

Feed costs constitute one of the largest expenditures incurred by livestock farmers. In Oman, the use of imported commercial feed is a burden on livestock producers. Therefore, this study aimed to lower feed costs by replacing commercial feed with an alternative feed mix that utilizes agricultural by-products readily available in Oman and assessing its effects on male Omani lambs. Twenty young male Omani lambs were divided into two equal groups: one group was fed a traditional commercial diet, and the other was fed an experimental diet of date palm fronds, barley, fish meal, date syrup, *Moringa oleifera* leaves, and salt. The results showed that both groups had similar weight gains at the end of the nine-week trial period, suggesting that the alternative feed performed just as effectively as the commercial feed, while saving 58% of the farmers on feed costs and 63 percent on cost per kg weight gain. Blood samples taken throughout the study confirmed that lambs fed the alternative feed had no health deficiencies and greater iron availability. Farmers can use this affordable alternative feed to reduce production costs, while keeping livestock healthy and producing efficient production.

## 1. Introduction

Feedstuff is by far the most expensive component in ruminant production chains, accounting for approximately 60–70% of the total costs [1,2]. Therefore, improving feeding strategies to enhance feed efficiency with no adverse effects on animal performance and health status is one of the main goals in livestock production to achieve sustainability and profitability in farming enterprises [3,4]. It is difficult to ensure a good balance between nutrient requirements, feed acceptance, economic feasibility, and ethical aspects of animal feeding without negative impacts on animal health and productive performance [5,6]. This is exacerbated in dry tropical areas, where the availability of conventional feed ingredients is scarce, feed resources are mostly seasonal, and the generation of agricultural residues is common [7,8].

Weaning is a vulnerable phase for youngstock and is usually associated with drastic physiological alterations, such as the switch from milk to dry feed consumption, increased maintenance requirements due to growth rate, the establishment of rumen fermentation characteristics, and the maturity of metabolic and immune functions [9,10]. The assessment of productive performance alone may not be adequate during this stage due to the high susceptibility to nutrient imbalance, production stress, and diseases [11]; therefore, determining the productive performance and physiological status of animals is vital when experimenting with dietary regimens. Rumen development and adaptation start before birth, with anatomically and functionally different characteristics, and continue after birth to develop into a functioning organ with microbial fermentation and absorption capabilities [12,13]. Rumen ontogeny offers an excellent opportunity to modulate the production and metabolic performance of young animals [14].

Non-conventional feed ingredients from agricultural and fishery by-products are potential ways to lower production costs and are at the heart of achieving zero-waste goals [15]. Date palm by-products consist of huge amounts of date palm fronds and seeds generated after harvest every year, which have low market value but have the potential to be included as a dietary supplement in ruminant diets [16]. Date palm fronds have been used as roughage for ruminants but have received less attention as a feed ingredient. *Moringa oleifera* is another supplement that is gaining momentum as an animal feed because of its crude protein content and richness of amino acids and other bioactive chemicals [17]. Meat and bone meal, hydrolyzed feather meal, and fish processing waste are examples of non-conventional feed ingredients from fishery by-products with highly digestible protein that can be used in livestock feeding [18].

This study aimed to evaluate an alternative cheap diet for growth performance, physiological status, and profit/cost analysis of post-weaning lambs. We predicted that supplementation with an alternative concentrate would decrease the cost/kg of gain compared to a conventional diet without any detrimental effects on the average daily gain, hematological profiles, and biochemical parameters of post-weaning lambs. Growth performance, hematological indices, serum biochemical parameters, and profit/cost analyses of the animals were evaluated.

## 2. Materials and Methods

### 2.1. Chemical Analysis of Feeds

Weekly samples from each dietary treatment were collected throughout the study and combined. Formulated concentrate was designed to be isonitrogenous and isoenergetic to commercial concentrate. The samples were analyzed in triplicate for dry matter (DM; method 930.15), crude protein (CP; method 984.13), ether extract (EE; method 920.39), and ash (method 942.05) using standard AOAC procedures [18]. Neutral detergent fiber (NDF) and acid detergent fiber (ADF) were determined according to Van Soest et al. [19], with heat-stable amylase and sodium sulfite added during the NDF procedure. Calcium and phosphorus levels were determined using atomic absorption spectrophotometry (PerkinElmer, Inc., Waltham, MA, USA) following acid digestion. GE was measured using a bomb calorimeter (Parr Instrument Company, Moline, IL, USA). All analyzed values were used for the statistical analyses; small differences between formulated and analyzed values are normal, as laboratory analysis produces more accurate estimates than values formulated by calculation. ME content was predicted using the equations given in [19] from chemical composition, according to the following:

ME (MJ/kg DM) = 0.015 × CP + 0.034 × EE + 0.014 × NFE + 0.010 × CF

where CP = crude protein, EE = ether extract, NFE = nitrogen-free extract, and CF = crude fiber, all expressed as g/kg DM.

### 2.2. Experimental Site, Animals, and Design

This feeding experiment was conducted at the Animal Production Center, Al-Rumais, Wilayat Barka, Sultanate of Oman (23.68° N, 57.99° E), from 17 April to 21 June 2025. The ambient temperature ranged between 28 °C and 35 °C during the experimental period. Twenty male Omani lambs with an initial body weight of 12.31 ± 3.22 kg and an average age (5 months) were used for this study. All lambs in this experiment were clinically healthy, vaccinated, and received antiparasitic medications to treat internal and external parasites prior to the start of the feeding trial. Animal care and use were performed in accordance with approved guidelines and protocols. The research protocol was reviewed and approved by the Animal Ethics and Professional Conduct Committee Ministry of Agriculture Fisheries Wealth and Water Resources, Sultanate of Oman (MAFWR-AHPC-AEC-2025-047).

Male lambs, 5 months old, were assigned to treatments using a completely randomized design. Baseline body weight was similar between groups (12.81 ± 3.75 vs. 11.81 ± 2.69 kg), suggesting that there was good balance at baseline. Animals were individually housed in pens with concrete floors (1.5 × 2.0 m) that had adequate airflow with minimal exposure to direct sunlight. Individual housing allowed for the accurate determination of feed intake and avoided competition for feed. However, it may cause temporary stress, leading to depressed intake while animals are adapting, as lambs are social animals. Fresh water and Rhodes grass hay were provided ad libitum throughout the experiment. After a 14-day adaptation period, during which animals were gradually adapted to the experimental diets from their previous diet, the 63-day experimental period began. Initial body weights were measured at the start of the adaptation period, on day 0 of the experimental period, and then every 14 days thereafter following an overnight fast (~16 h).

### 2.3. Dietary Treatments and Feeding Management

Lambs were allocated randomly to treatments, with no prior blocking, and the body weight of animals was recorded on arrival and used as a baseline value prior to the start of the feeding trial. Two dietary treatments were included in this study: (1) a control diet, which consisted of commercially available pelleted lamb fattening concentrate, and (2) a formulated concentrate. The commercial control diet was purchased from a local feed mill and was representative of a typical concentrate formulation that was fed to lambs grown under commercial farming practices in Oman. The diet was formulated to meet or exceed all nutrient requirements for growing lambs, as established by the National Research Council [20].

The formulated concentrate was composed of date palm fronds (29%), barley grains (20%), fish meal (12%), date syrup (10%), *Moringa oleifera* leaves and stems (28%), and salt (1%) on an as-fed basis. The date palm fronds were harvested from mature trees on local date palm farms. The trees were chopped to approximately 2–3 cm in length using a tree chopper machine (Fanda Machinery, Zhengzhou, China). After chopping, palm fronds were laid in the sun for 5–7 days to allow drying. After drying, they were stored in covered barns to protect them from rain and direct sunlight. *M. oleifera* stems and leaves were collected from wild growing trees and dried prior to their addition to the diet. Fish meal was sourced from local fish markets, sun-dried, and ground before incorporation into the diet. Barley grain was purchased from local suppliers and was of feed grade. Date syrup is a sweet concentrate obtained from dates that are unsuitable for human consumption. It had a Brix value of ~70–75. All the ingredients were mixed using a vertical mixer (Changzhou Farthest Machinery Co., Ltd., Changzhou, China).

The formulated concentrate was produced to provide similar crude protein and metabolizable energy as commercial concentrate to meet or exceed NRC nutrient recommendations [20] for growing lambs. Concentrate was formulated to be isonitrogenous and isoenergetic to commercial concentrate, as determined by laboratory analysis of individual ingredients. Chemical analysis of the formulated concentrate and commercial concentrate was conducted, and analyzed values were used for statistical purposes; small differences between formulated and analyzed values are expected, as laboratory analysis is more accurate. The chemical composition of the commercial concentrate was 13.45% CP and 11.2 MJ/kg ME on a DM basis, while for formulated concentrate it was 13.58% CP and 10.8 MJ/kg ME. Metabolizable energy was calculated from chemical composition using the equation of [19]. Lambs in both experimental groups were provided concentrate feed at 3.5% of their body weight (BW; dry matter basis), as recommended by the NRC [20], whereas Rhodes grass hay was offered ad libitum throughout the study period daily at 08:00 a.m. This level of feeding allowed the animals to eat ad libitum while minimizing feed waste. Feeds offered and refused were weighed separately on a daily basis for each animal. The refused feed was collected before the next feeding, weighed, and discarded. Feed intake data were collected and analyzed throughout the feeding experiment to determine the total intake, daily average feed intake, and patterns of feed intake over time.

### 2.4. Growth Performance Measurements

Animals were weighed on day 0 and every 14 days after overnight fasting (~16 h) throughout the feeding experiment. All weights were measured between 08:00 and 10:00 h. Animals were weighed on a calibrated scale (scale range 0–150 kg) with an accuracy of ±0.1 kg (Iconix FX1, Auckland, New Zealand). The total weight gain (BWG) was calculated by subtracting the final BW from the initial BW. The average daily gain (ADG) was calculated by dividing the total weight gain by the length of the experiment. Feed efficiency (FE) was calculated as the ratio of BWG to the total feed consumed (kg/kg). The feed conversion ratio (FCR) was calculated as the inverse of FE (kg feed/kg gain). These metrics were used to assess the growth performance.

### 2.5. Blood Sampling and Laboratory Analysis

Blood samples were obtained from the jugular veins of each animal at days 0 and 63 before feeding, to avoid postprandial modification of blood variables. Approximately 10 mL of blood was collected into K2EDTA evacuated tubes from the jugular vein for hematology analysis and 5 mL into non-additive tubes for serum biochemistry analysis. Blood samples were analyzed within 2 h using an automated veterinary hematology analyzer (SYSMEX pocH-100iV, Sysmex Corporation, Kobe, Japan). The following hematological parameters were determined: red blood cell count (RBC), hemoglobin concentration (Hgb), hematocrit (Hct), mean corpuscular volume (MCV), mean corpuscular hemoglobin (MCH), mean corpuscular hemoglobin concentration (MCHC), red cell distribution width standard deviation (RDW-SD), red cell distribution width-coefficient of variation (RDW-CV), white blood cell count (WBC), leukocyte differentials, platelet count, nucleated red blood cell count (NRBC), reticulocyte %, reticulocyte hemoglobin equivalent (Ret-He), immature reticulocyte fraction (IRF), and low fluorescence ratio (LFR). For serum biochemistry, blood in serum separator tubes was left to clot for 30 min at room temperature and centrifuged at 3000× *g* for 15 min. Serum was pipetted into cryovials and frozen at −20 °C until analysis. Serum was analyzed for glucose, total protein, albumin, blood urea nitrogen (BUN), creatinine, calcium, phosphorus, and copper using commercial diagnostic kits (RANDOX Laboratories Ltd., Crumlin, UK) and a semi-automated biochemistry analyzer (Humalyzer 3000, Human Diagnostics, Wiesbaden, Germany).

### 2.6. Economic Analysis

An economic analysis was conducted to determine the production cost and economic conversion ratio of animals fed both diets. The feed costs were determined based on the current market price in Oman during the experimental period. The cost of the commercial concentrate was priced at 150 OMR/ton (~388 USD/ton). The ingredient costs for the formulated concentrate were as follows: date palm fronds, 20 OMR/ton; fish meal, 300 OMR/ton; barley grain, 120 OMR/ton; date syrup, 80 OMR/ton, *Moringa oleifera*, 50 OMR/ton; and salt, 50 OMR/ton. The average cost of the formulated concentrate is calculated to be 52.4 OMR/ton. The total feed cost per animal was calculated by multiplying the total feed consumed by the total feed cost. The cost of gain was calculated by dividing the total feed cost by the total BWG. The economic conversion ratio was calculated by dividing the total feed cost by the BWG.

### 2.7. Statistical Analysis

Statistical analyses were performed using IBM SPSS Statistics version 21.0 (IBM Corp., Armonk, NY, USA). Growth performance, feed intake data, feed efficiency, and economics were analyzed using an independent sample *t*-test. Hematological parameters and biochemical variables were analyzed using two-way repeated-measures analysis of variance (ANOVA), with time (day 0 vs. day 63) as the within-subject factor and diet as the between-subject factor. Sphericity was determined using Mauchly’s test with the Greenhouse–Geisser correction applied when appropriate. Significant time × diet interactions were followed with a simple main-effect analysis. Weekly concentrate intake was analyzed using repeated-measures ANOVA with week as the within-subject factor, while baseline concentrate intake was included as a covariate to account for initial variability among lambs. The effect size was calculated using partial eta squared (η^2^) and Cohen’s d for the ANOVA and *t*-tests, respectively. α was set at 0.05. Data are presented as the mean ± standard deviation unless stated otherwise. The assumptions for parametric analyses were tested and met. Normality was tested using the Shapiro–Wilk test, and homogeneity of variance was tested using Levene’s test.

## 3. Results

### 3.1. Feed Composition and Nutritional Analysis

The mean values of the chemical composition of the ingredients of the formulated concentrate are presented in Table 1.

Dry matter values ranged from 96% in salt to 93.03% and 92.81% in *Moringa oleifera* and waste fish meal, respectively, while it was 56% in date syrup. The protein content of the ingredients included in the formulated concentrate was different, varying from 1.2% in date syrup to 42.5% in waste fish meal and 20.85% in *Moringa oleifera*. The ether extract content of the ingredients was highest in waste fish meal (15.25%) and lowest in date syrup (0%), followed by *Moringa oleifera* (4.15%) and date palm fronds (4.78%).

The fiber fractions also varied widely between ingredients, with neutral detergent fiber being high in *Moringa oleifera* and date palm fronds (48% and 48.6%, respectively), while it was 23% and 20% in waste fish meal and barley, respectively. Acid detergent fiber content ranged from 3.6% in waste fish meal to 39.8% in date palm fronds. The hemicellulose content of the ingredients ranged from 8.8% in date palm fronds to 19.4% in fish waste. Ash values were high in waste fish meal (28.52%) and *Moringa oleifera* (14.35%), while the lowest values were in barley (2.6%). Gross energy (GE) values in ingredients ranged from 12.93 MJ/kg in barley to 19.83 MJ/kg in date palm fronds. *Moringa oleifera* and waste fish meal had intermediate values of GE (14.37 and 13.94 MJ/kg, respectively). The percentage values of calcium and phosphorus were highest in waste fish meal (5.36 and 3.36%, respectively), followed by *Moringa oleifera* (2.427 and 0.244%, respectively).

Chemical analysis of the experimental diets revealed that both diets had similar crude protein and metabolizable energy contents, as shown in Table 2. The control diet (commercial concentrate) contained 95.02% DM, 13.45% CP, 4.32% EE, 38.25% NDF, 20.66% ADF, 11.96% ash, 1.12% calcium, 0.62% phosphorus and 10.98 MJ/kg ME. The formulated concentrate had 93.32% DM, 13.58% CP, 3.91% EE, 36.22% NDF, 21.97% ADF, 11.64% ash, 1.14% calcium, 0.54% phosphorus and 10.67 MJ/kg ME on a DM basis.

### 3.2. Body Weight Gain and Feed Intake

**Figure 1:**
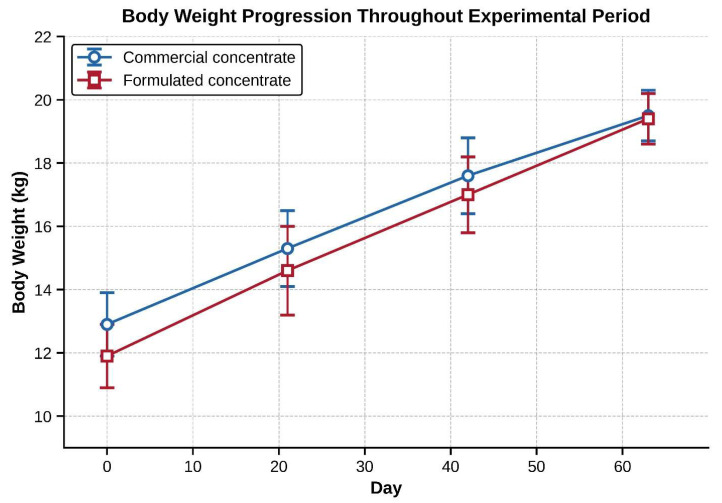
Body weight gain (kg) for control and treatment groups from day 0 to day 63. Values represent mean ± SE. Both groups showed similar growth trajectories (*p* = 0.864 for final weights).

There were no significant differences in BW, BWG, or feed intake among the groups (Table 3 and Figure 1). Basal and final BWs were not significantly different among the groups (basal BW: 12.81 ± 3.75 kg vs. 11.81 ± 2.69 kg, *p* = 0.502; final BW: 19.56 ± 3.09 kg vs. 19.33 ± 2.81 kg, *p* = 0.864), indicating that the animals were well randomized. The mean BWG was similar between the groups (*p* = 0.322). However, the BWG was higher (11.4%) in the treatment group than in the control group, though not significantly (7.52 ± 1.52 kg vs. 6.75 ± 1.84 kg).

### 3.3. Weekly Feed Intake

**Figure 2:**
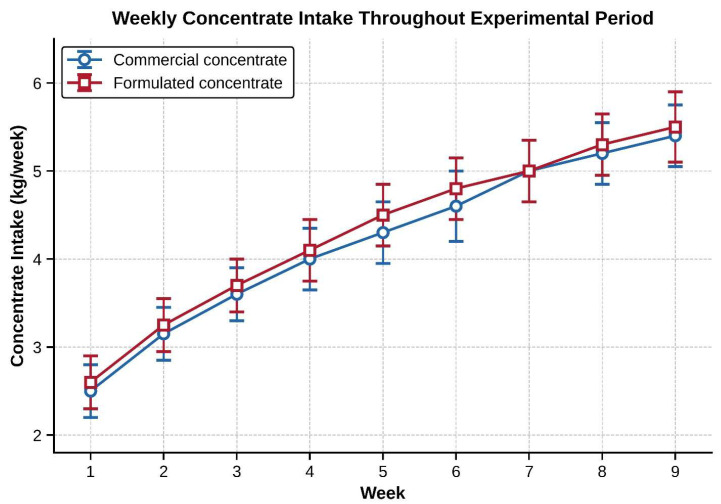
Weekly concentrate intake (kg) for control and formulated concentrate groups throughout the 63-day experimental period. Values represent mean ± SE. No significant time × group interaction was observed (*p* = 0.257), indicating parallel development of feeding behavior and comparable palatability.

But time × diet interaction (*p* = 0.257), indicating the normal development of feeding behavior in both groups (Figure 2). Concentrate intake increased steadily from approximately 2.5 kg during week 1 to 5.5 kg in week 9, as expected for healthy animals after weaning, which increased their dry matter intake capacity as rumen volume and fermentation capacity increased. Similar intake patterns between groups suggested that the alternative formulation was well accepted and palatable, with no indication that lambs would refuse feed or eat less due to unpalatability.

### 3.4. Hematological Parameters

**Figure 3:**
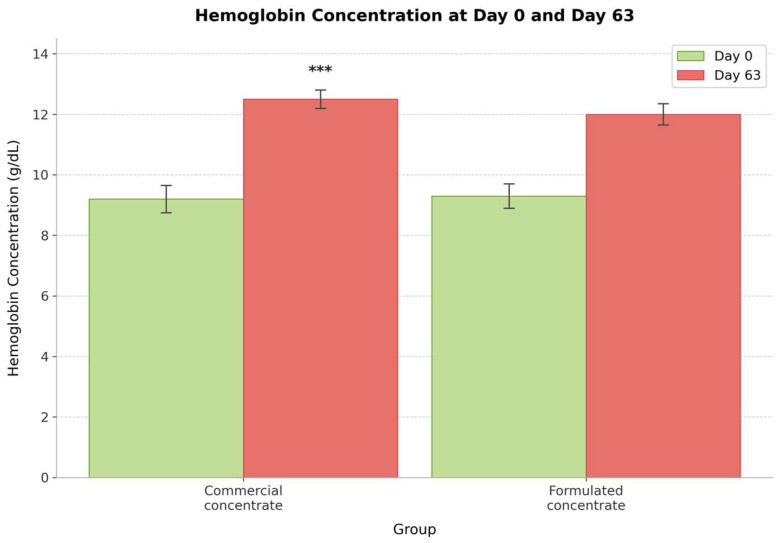
Hemoglobin concentration (g/dL) at day 0 and day 63 for both experimental groups. Both groups showed significant increases over time (*p* < 0.001), with a significant time × diet interaction (*p* = 0.025). *** *p* < 0.001.

Repeated-measures ANOVA detected time-course changes and treatment effects on hematological parameters (Table 4, Figure 3). There was a significant time × diet interaction for red blood cell count (F (1,18) = 6.45, *p* = 0.016), suggesting that the groups changed differently over time. Counts of red blood cells increased significantly in both groups (control: 10.20 ± 2.36 to 12.48 ± 1.82 × 10^12^/L; formulated concentrate: 9.85 ± 1.44 to 12.56 ± 1.20 × 10^12^/L).

### 3.5. Serum Biochemistry

Serum biochemical parameters are summarized in Table 5. Diet had no effect (*p* > 0.05) on any parameter, suggesting that the formulated concentrate was metabolically similar to the commercial concentrate. However, there was a significant time effect on glucose (*p* < 0.001) and triglycerides (*p* = 0.006). Glucose concentrations decreased from day 0 to day 63 in both the commercial (65.80 ± 2.63 vs. 55.40 ± 2.45 mg/dL) and formulated (73.00 ± 2.63 vs. 58.20 ± 2.45 mg/dL) treatment groups. Diet had a significant effect on glucose concentrations (*p* = 0.038); however, because there was no significant time × diet interaction (*p* = 0.208), the pattern of change over time was similar between groups. There were no significant time, diet or time × diet effects on serum albumin, globulin, total protein, urea, creatinine, AST, ALT, or ALP (*p* > 0.05 for all parameters), and all values fell within normal physiological ranges throughout the experimental period.

### 3.6. Economic Performance Indicators

**Figure 4:**
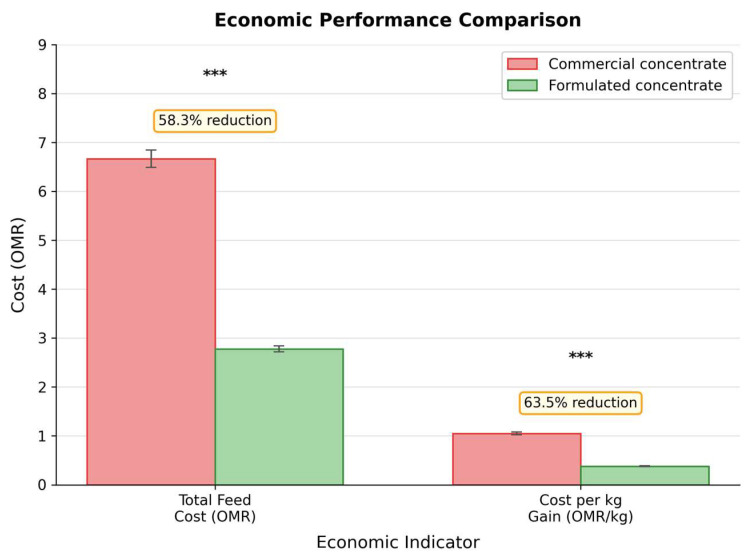
Comparative economic performance showing total feed cost and cost per kg gain for control vs. treatment groups. Treatment group demonstrated 58.3% reduction in total feed costs and 63.5% reduction in cost per BWG kg (*** *p* < 0.001).

There were statistically significant differences in favor of the treatment group for all the economic variables analyzed (Table 6, Figure 4). Concentrate cost was 58.3% lower (2.05 ± 0.22 vs. 5.87 ± 0.96 OMR; *p* < 0.001; Cohen’s d = 5.02) in the treatment group (Figure 4), which can be considered a very large effect size. There was a 58.3% lower feed cost in the treatment group (2.77 ± 0.33 vs. 6.64 ± 1.01 OMR; *p* < 0.001; Cohen’s d = 4.86). Lastly, cost per kg BWG was 63.5% lower in the treatment group (0.38 ± 0.07 vs. 1.04 ± 0.30 OMR/kg BWG; *p* < 0.001; Cohen’s d = 2.73).

## 4. Discussion

### 4.1. Chemical Composition of Feeds

The chemical composition of the diets indicated that both the commercial concentrate and formulated concentrate met the nutritional requirements for growing lambs. Nutrient concentrations were 13.45% CP and 10.98 MJ/kg ME for commercial concentrate and 13.58% CP and 10.67 MJ/kg ME for formulated concentrate, falling within the recommended range for the optimal growth performance of post-weaning lambs [20]. Adequate nutrient density is important during this phase because protein synthesis rates and energy requirements increase in growing ruminants [21].

The higher levels of NDF in the formulated concentrate were a result of the addition of date palm fronds, which are a fiber source [16,22]. Date palm fronds are regarded as agricultural waste because they are rarely used by farmers for any purpose. However, date palm fronds are rich in structural carbohydrates that aid rumen function and motility. Both NDF values fell below 30–40%, which is considered too high for digestibility and voluntary intake of concentrate diets [23]. This is very important in young ruminants because high-fiber diets may limit animal performance by reducing energy intake, owing to incomplete rumen development [24,25].

The Ca:P ratio was within the recommended level of 1.5:2.0:1 in the commercial concentrate (1.81:1) and formulated concentrate (1.84:1) diets [26]. High levels of calcium compared to phosphorus have been linked to metabolic diseases, reduced bone mineralization, and decreased animal performance [27]. The experimental diets achieved a Ca:P ratio below 2:1 despite utilizing unconventional feed ingredients, which shows that agricultural by-products can be mixed strategically to hit targeted mineral levels.

*Moringa oleifera* leaves were included at a 28% inclusion rate in the formulated concentrate diet. *Moringa* leaves are rich in protein [28,29]. Crude protein content ranged between 25 and 30% on a dry matter basis. *Moringa oleifera* leaves also contain amino acids such as lysine, methionine, and threonine, which are essential for animals [17]. The leaves contain β-carotene (25 mg/100 g fresh weight), vitamins C and E, and other bioactive compounds, such as flavonoids, phenolic acids, and glucosinolates, which have antioxidant and antimicrobial properties [28,30].

Fish meal constituted 12% of the formulated concentrate diet. Fish meal is a highly digestible protein source with a balanced amino acid profile. Fish meal is also a source of highly digestible essential fatty acids, such as EPA and DHA. Fish meal also contains readily absorbed minerals such as iron, zinc, and selenium [31,32]. It has been shown to improve protein utilization through a balanced amino acid profile at the intestinal level in young ruminants, which still have a developing rumen [33]. Fish meal can provide omega-3 fatty acid benefits such as anti-inflammatory responses and improved immunity during the transition period.

Date syrup constituted 10% of the formulated concentrate diet. Date syrup contains readily fermentable carbohydrates, making it highly palatable and providing readily available energy for microbes and the host [16]. Date syrup is highly concentrated in sugar; it contains approximately 70–80% sugar on a dry matter basis. Date syrup can improve rumen fermentation and volatile fatty acid production. It also improves palatability, which can lead to improved voluntary intake among young ruminants weaned off milk-to-solid diets [34]. Date syrup provides essential minerals, such as potassium, magnesium, and iron.

Locally available agricultural by-products matched the nutrient composition of commercial concentrates. Agricultural by-products can be used to partially or completely replace commercial feeds. This will reduce the environmental waste deposited by these agro-industries. The use of agricultural by-products will help improve the economic sustainability of livestock production, especially in areas where traditional ingredients are limited or unavailable, owing to seasonal production or high market prices [2,31]. Sheep production in arid and semi-arid regions faces many feed challenges due to seasonal production and the high prices of commonly used feeds.

### 4.2. Growth Performance and Feed Efficiency

There was no difference in growth performance between the commercial concentrate- and the formulated concentrate-fed lambs. The total weight gain (7.52 ± 1.52 vs. 6.75 ± 1.84 kg, *p* = 0.322) and average daily gain (0.119 ± 0.024 vs. 0.107 ± 0.029 kg/day, *p* = 0.322) were similar between animals on the formulated concentrate diet and animals on the commercial concentrate diet. This was expected because animal performance is highly affected by nutrient density and not by ingredient quality or source [35,36].

Our findings were within the reported average daily gain of hair sheep breeds during the post-weaning stage. The average daily gain of Barbary sheep under an intensive management system ranged from 0.095 to 0.140 kg/day [37]. The average daily gain reported by Al-Khalasi et al. for Omani sheep was 0.117 kg/day [38]. Our findings fell within this range, which suggests that our diets supported the normal growth and development of the animals used in this study.

Our study successfully overcame the challenge of using 28% moringa leaves as an inclusion in ruminant diets. Many studies have reported poor feed intake when moringa leaves were included in ruminant diets above the threshold level, owing to the presence of glucosinolates in moringa leaves. The consumption of feed containing moringa did not differ from that of commercial concentrate-fed animals. The 28% inclusion rate used in this study did not exceed the threshold level [17].

Feed conversion was not different between commercial concentrate- and formulated concentrate-fed lambs (5.88 ± 0.72 vs. 6.30 ± 0.85 kg feed/kg gain, *p* = 0.254). These values suggest that it took approximately 6 kg of concentrate to gain 1 kg of body weight. Feed conversion was slightly lower for animals receiving the formulated concentrate, but differences were not significant. This suggests that animals fed a formulated concentrate diet utilized nutrients better than those fed a commercial concentrate diet.

This transition period is crucial for animals. This is the time they wean from milk and slowly adapt to roughage [39]. They undergo many metabolic, immunological, and digestive changes during this phase, which makes them susceptible to disease and poor performance. The increase in concentrate intake was gradual in the animals included in the study. This might be due to an increase in rumen capacity as they adapt to solid diets [40]. There were no signs of digestive upset or poor weight gain among the animals. This suggests that the nutrients were balanced and the ingredients were compatible.

The date palm fronds constituted 29% of the formulated concentrate diet. Date palm fronds can be considered low-quality roughages because of their high lignin content [23]. Despite this, the nutritional requirements of ruminants can still be met when agricultural by-products are appropriately processed and used strategically in the diet. Animals on the formulated concentrate diet received fibers from date palm fronds that met the fiber requirements without compromising the energy density needed for growth. Najar et al. [41] reported that processed materials from date palms can replace 20–25% of roughage without affecting animal performance.

### 4.3. Hematological Status and Iron Nutrition

Both the commercial concentrate and formulated concentrate improved the RBC, HGB, and HCT, which was expected because animals experience an increase in these parameters during the rapid growth phase after weaning [42,43,44]. However, this increase was greater in the formulated concentrate-fed lambs than in the commercial concentrate-fed lambs. This effect may be associated with the high bioavailability of iron in fish meal, which contains heme iron, as reported previously [40]; however, iron form and absorption were not directly evaluated in the present study. The absorption rate of heme iron ranges from 15 to 35%, whereas non-heme iron absorption ranges from 2 to 20% [45]. The post-weaning stage is usually associated with an increased iron requirement. Young animals need more iron to help with hemoglobin synthesis to meet the demand for increasing blood volume and myoglobin for muscle deposition.

Ret-He increases when more iron is available for erythropoiesis [46]. The increase was significant in the formulated concentrate-fed lambs (*p* = 0.018) compared with the commercial concentrate-fed lambs. This shows that the formulated concentrate diet had more bioavailable iron than the commercial concentrate diet did. Ret-He has been used as an indicator of iron status, which is more sensitive and responds rapidly to changes in iron status compared to traditional iron status indicators, such as serum ferritin concentration and total iron-binding capacity [47]. A significant increase in Ret-He showed that both diets had sufficient bioavailable iron to support the increased demand for post-weaning lambs. A better improvement was observed in the formulated concentrate-fed lambs, owing to the inclusion of fish meal.

Iron deficiency is common in young growing ruminants, especially in intensive production systems in which milk feeding is limited [43,48]. Failure to provide adequate iron to meet demand affects the oxygen transport capacity, immunity, and overall performance of the animal through reduced feed intake, protein synthesis, and oxidative metabolism [49]. Both diets supported normal hematopoiesis because there was no difference in the hemoglobin parameters of the commercial concentrate- and formulated concentrate-fed lambs at the end of the study. Both diets provided adequate iron to support the increased need for growing lambs after weaning.

The decrease in RDW-SD was significant (13–15%, *p* = 0.024). An increase in RDW values has been linked to iron deficiency or any other nutritional deficiency that can affect uniform hemoglobin synthesis [50]. A decrease in RDW resulted in an improvement in the iron status of the animals fed both diets. Improvement in hematological status was shown by a parallel reduction in RDW-SD accompanied by no change in MCV and MCH values [51]. This shows that animals receive adequate iron from their diets as they adapt to their post-weaning diets.

The platelet counts increased; however, there was a significant time × diet interaction (*p* < 0.001). Platelet production can be affected by multiple micronutrients, such as vitamin B12, folate, and iron [52]. The absence of vitamin B12, folate, or iron results in poor platelet production. The pattern of increase showed that animals fed the formulated concentrate might have had better micronutrient status or availability compared to animals fed a commercial concentrate diet. Although this was not directly measured, the results from this study suggest that the better performance of formulated concentrate-fed lambs might have resulted from better micronutrient status.

White blood cells did not differ between commercial concentrate- and formulated concentrate-fed lambs throughout the study. This suggests that both diets support normal immunity among animals. Leukocytosis or leukopenia was not observed. The safety of moringa was demonstrated because there were no effects on white blood cell counts, even though animals were fed diets containing 28% *moringa* [53]. Many in vitro and in vivo reports have shown the beneficial effects of moringa, such as increased immunoglobulin production and improved cell-mediated immunity [54]. *Moringa* had no negative effects on the animals used in this study.

### 4.4. Metabolic and Biochemical Adaptations

There was no diet effect for any of the serum biochemical variables analyzed (*p* > 0.05), indicating nutritional equivalency between the two experimental diets used in this trial. Homeostasis was likely maintained for these physiological variables across the feeding period in both dietary treatments. It has been reported previously that balanced energy and crude protein diets elicit similar responses at the systemic level in small ruminants [55,56]. The sheep offered two experimental diets had no significant differences in most blood biochemical indicators, including AST, glucose, total protein, ALT, triglycerides, and creatinine concentrations [57].

Serum glucose concentration was affected by time (*p* < 0.001). Glucose values decreased from day 0 to day 63 in both the commercial (65.80 ± 2.63 vs. 55.40 ± 2.45 mg/dL) and formulated (73.00 ± 2.63 vs. 58.20 ± 2.45 mg/dL) groups. There was a diet effect for glucose (*p* = 0.038) but no interaction between time and diet was observed (*p* = 0.208); therefore, both groups trended similarly over time without any clinically significant differences. The endogenous source of glucose in ruminants is through gluconeogenesis in the liver and is largely attributed to propionate [58]. Serum glucose concentration is also largely affected by the duration of the feeding period as rumen function and fermentation processes become more adapted to the diet [59]. The observed decrease in glucose across the feeding period could be explained by increased peripheral uptake of glucose for deposition and animal growth as fattening continued [60]. Glucose concentrations remained within expected reference ranges throughout the trial [61,62].

Time affected triglyceride concentration (*p* = 0.006). This is not unexpected, as mobilization and subsequent deposition of triglycerides is expected to occur during periods of active growth, which would lead to changes in serum triglyceride concentrations [63,64]. The diets had no effect on triglycerides (*p* = 0.762).

Diet, time, and diet × time had no effect on serum albumin, globulin, total protein, urea, creatinine, AST, ALT or ALP concentrations (*p* > 0.05), and all values fell within normal physiological ranges. Activities of liver enzymes were not altered between groups, indicating that no hepatocellular damage occurred due to formulated concentrate [63]. Serum albumin and total protein concentrations indicated adequate protein intake and hepatic synthetic ability in both experimental diet groups [57,62].

### 4.5. Economic Feasibility and Environmental Impact

The significant reduction in cost per kg gain (63%) and total feed cost (58%) associated with the alternative diet was substantially higher than the previously reported averages of 25–45% when testing new feed ingredients [65]. This improvement can likely be attributed to the combination of several factors, such as minimal transport distances by using local ingredients, inclusion of waste by-products with little to no commercial value, and the elimination of expensive commercial feed ingredients when possible without sacrificing diet quality [43].

Using agro-industrial by-products also benefits producers by reducing the costs associated with disposal and taking advantage of low-cost or free feed sources [64]. This philosophy applies to date palm fronds, which are typically disposed of by burning during large-scale annual pruning events [16]. Additionally, moringa leaves could be valorized as an ingredient where moringa is produced for seed extraction [29].

Feed expenses account for roughly 60–70% of total sheep production costs; thus, diet changes that result in savings can significantly improve the bottom line for producers [65]. Locally sourced ingredients are less susceptible to global market disruptions or price fluctuations in conventional feeds while promoting regional economies [17].

### 4.6. Further Study Recommendations

Research measuring rumen fermentation parameters, such as VFA production, ruminal NH3-N, and rumen microbial counts, would be beneficial to confirm whether the similar performance observed between diets was accompanied by similar fermentation patterns or if different patterns occurred with compensatory mechanisms in place to maintain animal performance. Thus, we suggest conducting a controlled metabolic trial to accurately measure DMI, CPI and MEI and to determine apparent nutrient digestibility coefficients under predefined conditions.

Additionally, future studies could analyze carcass characteristics, meat quality parameters, and sensory analysis to determine whether cheaper dietary formulations produce meat of equal quality. Fish meal was included in the formulated concentrate; therefore, fish oil could have affected meat quality through altered fatty acid deposition. Assessing the consumer acceptance of meat from animals fed these diets could also be analyzed to determine whether there is a marketable difference. Including a complete economic analysis of production and consumers would provide useful information for producers when deciding whether a diet should be utilized.

It would be interesting to test this diet on a larger scale and at multiple locations to assess the scalability of this study. The inclusion of multiple breeds and production systems would allow this diet to be tested in a wide variety of environments. Long-term studies that follow animals from weaning to finishing would be able to analyze the long-term effects of diet and cumulative benefits.

## 5. Conclusions

The findings of this research demonstrate that feed costs can be reduced by incorporating locally available, low-cost ingredients. The formulated diet containing date palm fronds (29%), waste fish meal (12%), barley grain (20%), date syrup (10%), and *Moringa oleifera* leaf meal (28%) reduced the cost of the diet by 63% compared to the control diet. This resulted in similar BWG (7.52 ± 1.52 vs. 6.75 ± 1.84 kg, *p* = 0.322), trends for improved ATFI-adjusted FCR (5.88 ± 0.72 vs. 6.30 ± 0.85, *p* = 0.254) and had no effect on eating behavior. Health was not affected by dietary treatment, as shown by adaptive increases in RBC, hemoglobin, and hematocrit concentrations (*p* < 0.025), and improved RHC, suggesting sufficient iron availability (*p* = 0.018), and no significant differences in BUN or creatinine. This diet decreased the concentrate cost by 65%, total feed cost by 58%, and increased the ERC by 63.5% compared to the control diet (all *p* < 0.001). As feed accounts for 60–70% of the total sheep production costs, these results could allow for an approximate 38% reduction in total production costs. By including agro-industrial by-products in animal diets, we can improve the overall carbon footprint of agriculture and help alleviate some of the waste management issues that arise from disposal.

## Figures and Tables

**Table 1:** Chemical composition of feed ingredients used in formulated total mixed ration (% dry matter basis unless otherwise stated).

Component	*Moringa oleifera*	Barley	Date Syrup	Date Palm Fronds	Fish Meal	Salt
Dry Matter (%)	93.03	89.50	56.00	91.20	92.81	96.00
Proximate composition (%DM)						
Crude Protein	20.85	10.50	1.20	4.20	42.50	0.00
Ether Extract	4.15	2.10	0.00	4.78	15.25	0.00
Ash	14.35	2.60	3.50	12.80	28.52	100.00
Nitrogen-Free Extract	32.65	64.80	95.30	30.42	10.73	0.00
Gross Energy (MJ/kg DM)	14.37	12.92	16.54	19.82	13.95	0.00
Fiber fractions (%DM)						
NDF	48.00	20.00	0.00	48.60	23.00	0.00
ADF	35.20	5.80	0.00	39.80	3.60	0.00
Hemicellulose	12.80	14.20	0.00	8.80	19.40	0.00
Cellulose	28.50	4.90	0.00	31.20	2.80	0.00
Lignin	6.70	0.90	0.00	8.60	0.80	0.00
Minerals (%DM)						
Calcium	2.427	0.050	0.020	0.650	5.360	0.000
Phosphorus	0.244	0.380	0.010	0.080	3.360	0.000
Ca:P ratio	9.95:1	0.13:1	2:1	8.13:1	1.60:1	—

NDF = neutral detergent fiber; ADF = acid detergent fiber; DM = dry matter; Ca:P = calcium-to-phosphorus ratio.

**Table 2:** Chemical composition and nutritional characteristics of commercial and formulated concentrates (% dry matter basis unless otherwise stated).

Component	Commercial Concentrate	Formulated Concentrate
Dry Matter (%)	95.02	93.32
Crude Protein	13.45	13.58
Ether Extract	4.32	3.91
Ash	11.96	11.64
NDF	38.25	36.22
ADF	20.66	21.97
Hemicellulose	17.59	14.25
Gross Energy (MJ/kg DM)	17.39	17.47
ME (MJ/kg DM)	10.98	10.67
Calcium	1.12	1.14
Phosphorus	0.62	0.61
Ca:P ratio	1.81:1	1.86:1

**Table 3:** Growth performance and feed efficiency parameters of lambs fed experimental diets.

Parameter	Commercial Concentrate (CC)	Formulated Concentrate (FC)	*p*-Value
Initial Body Weight (kg)	12.81 ± 3.75	11.81 ± 2.69	0.502
Final Body Weight (kg)	19.56 ± 3.09	19.33 ± 2.81	0.864
Total Weight Gain (kg)	6.75 ± 1.84	7.52 ± 1.52	0.322
Average Daily Gain (kg/d)	0.107 ± 0.029	0.119 ± 0.024	0.322
Feed Conversion Ratio	6.30 ± 0.85	5.88 ± 0.72	0.254
Feed Efficiency (kg gain/kg feed)	0.163 ± 0.022	0.172 ± 0.020	0.254
Total Concentrate Intake (kg)	39.11 ± 6.39	40.94 ± 4.41	0.466
Total Rhodes grass intake (kg)	7.77 ± 1.57	7.14 ± 1.63	0.391
Total Feed Intake (kg)	46.88 ± 6.92	48.08 ± 5.31	0.669
Daily Feed Intake (kg/d)	0.781 ± 0.115	0.801 ± 0.088	0.698

Values are presented as the mean ± SD. Statistical comparisons were performed using independent *t*-tests.

**Table 4:** Hematological parameters of lambs at the beginning and end of the experimental period.

Parameter	Unit	Commercial Concentrate (CC)		Formulated Concentrate (FC)		*p*-Value		
		Day 0	Day 63	Day 0	Day 63	Time	Diet	Diet × Time
WBC	×10^9^/L	12.41 ± 4.49	10.84 ± 3.15	11.08 ± 2.70	12.27 ± 3.02	0.828	0.970	0.128
RBC	×10^12^/L	10.20 ± 2.36	12.48 ± 1.82	9.85 ± 1.44	12.56 ± 1.20	<0.001 ***	0.845	0.597
HGB	g/dL	9.22 ± 1.88	12.55 ± 1.32	9.22 ± 1.33	12.05 ± 0.56	<0.001 ***	0.613	0.502
HCT	%	30.01 ± 6.23	39.33 ± 4.39	29.07 ± 4.41	37.24 ± 2.02	<0.001 ***	0.350	0.654
MCV	fL	29.85 ± 3.27	31.70 ± 1.94	29.51 ± 1.74	29.80 ± 2.25	0.055	0.242	0.152
MCH	pg	9.19 ± 1.11	10.15 ± 0.75	9.37 ± 0.40	9.65 ± 0.62	0.003 **	0.590	0.073
MCHC	g/dL	28.03 ± 8.93	31.98 ± 1.77	31.79 ± 1.08	29.57 ± 9.35	0.679	0.749	0.151
PLT	×10^9^/L	474.50 ± 311.26	576.80 ± 123.86	405.60 ± 225.62	515.00 ± 100.63	0.116	0.345	0.956
RDW-SD	fL	25.30 ± 3.35	21.94 ± 1.89	23.36 ± 4.72	21.36 ± 1.18	0.018 *	0.193	0.516
RDW-CV	%	27.50 ± 3.40	26.17 ± 2.38	26.43 ± 3.60	27.17 ± 2.35	0.870	0.977	0.096
NRBC	×10^9^/L	0.018 ± 0.012	0.027 ± 0.019	0.019 ± 0.020	0.012 ± 0.006	0.797	0.247	0.001 **
NEUT	×10^9^/L	2.86 ± 3.33	2.64 ± 2.19	1.93 ± 2.01	3.96 ± 2.07	0.163	0.831	0.087
LYMPH	×10^9^/L	7.62 ± 4.07	6.86 ± 3.86	7.29 ± 3.23	6.94 ± 2.27	0.488	0.924	0.402
MONO	×10^9^/L	1.27 ± 0.79	0.71 ± 0.27	1.09 ± 0.21	0.65 ± 0.19	0.001 **	0.431	0.039 *
EO	×10^9^/L	0.47 ± 0.27	0.49 ± 0.30	0.56 ± 0.39	0.57 ± 0.61	0.002 **	0.604	<0.001 ***
BASO	×10^9^/L	0.19 ± 0.08	0.14 ± 0.07	0.22 ± 0.17	0.13 ± 0.05	0.050	0.825	0.004 **
RET	%	0.41 ± 0.68	0.11 ± 0.20	0.08 ± 0.07	0.02 ± 0.03	0.335	0.073	0.144
IRF	%	20.74 ± 40.89	1.99 ± 2.66	2.39 ± 2.31	3.45 ± 4.61	0.187	0.218	0.142
LFR	%	79.26 ± 40.89	98.01 ± 2.66	97.60 ± 2.31	96.55 ± 4.61	0.187	0.218	0.142
MFR	%	0.95 ± 1.21	0.83 ± 1.67	2.23 ± 2.24	1.05 ± 2.95	0.312	0.307	0.408
HFR	%	2.47 ± 4.87	1.16 ± 1.53	0.13 ± 0.28	2.40 ± 3.41	0.634	0.570	0.088
Ret-He	pg	11.31 ± 0.65	12.77 ± 0.71	12.29 ± 0.40	12.50 ± 0.19	<0.001 ***	0.061	0.001 **

Values are presented as the mean ± SD. Statistical analysis was performed using repeated-measures ANOVA. * *p* < 0.05, ** *p* < 0.01, *** *p* < 0.001. RBC, red blood cell; MCV, mean corpuscular volume; MCH, mean corpuscular hemoglobin; MCHC, mean corpuscular hemoglobin concentration; RDW-SD, red cell distribution width standard deviation; Ret-He, reticulocyte hemoglobin equivalent.

**Table 5:** Serum biochemical parameters of lambs at the beginning and end of the experimental period.

Parameter	Diet Group	Day 0 (Mean ± SE)	Day 63 (Mean ± SE)	*p*-Value (Time)	*p*-Value (Diet)	*p*-Value (Time × Diet)
Albumin (g/dL)	Commercial	2.50 ± 0.11	2.68 ± 0.09	0.052	0.449	0.380
	Formulated	2.57 ± 0.11	2.63 ± 0.09			
Globulin (g/dL)	Commercial	3.51 ± 0.16	3.49 ± 0.14	0.941	0.728	0.941
	Formulated	3.58 ± 0.16	3.56 ± 0.14			
Total Protein (g/dL)	Commercial	6.01 ± 0.19	6.17 ± 0.15	0.366	0.518	0.704
	Formulated	6.15 ± 0.19	6.20 ± 0.15			
Glucose (mg/dL)	Commercial	65.80 ± 2.63	55.40 ± 2.45	<0.001 **	0.038 *	0.208
	Formulated	73.00 ± 2.63	58.20 ± 2.45			
Urea (mg/dL)	Commercial	37.90 ± 2.19	36.40 ± 2.15	0.463	0.457	0.730
	Formulated	36.00 ± 2.19	35.50 ± 2.15			
Creatinine (mg/dL)	Commercial	1.15 ± 0.04	1.12 ± 0.04	0.504	0.386	0.812
	Formulated	1.09 ± 0.04	1.07 ± 0.04			
Cholesterol (mg/dL)	Commercial	62.70 ± 2.67	64.60 ± 3.42	0.815	0.428	0.312
	Formulated	60.10 ± 2.67	68.40 ± 3.42			
Triglycerides (mg/dL)	Commercial	25.10 ± 1.55	27.50 ± 1.25	0.006 **	0.372	0.454
	Formulated	26.60 ± 1.55	28.10 ± 1.25			
AST (U/L)	Commercial	90.70 ± 4.28	91.20 ± 3.65	0.871	0.407	0.796
	Formulated	95.70 ± 4.28	95.40 ± 3.65			
ALT (U/L)	Commercial	28.50 ± 1.48	27.90 ± 1.48	0.444	0.369	0.758
	Formulated	26.90 ± 1.48	26.70 ± 1.48			
ALP (U/L)	Commercial	145.40 ± 8.12	148.60 ± 7.94	0.574	0.395	0.819
	Formulated	137.20 ± 8.12	138.90 ± 7.94			

Values are presented as the mean ± SD. Statistical analysis was performed using repeated-measures ANOVA. * *p* < 0.05, ** *p* < 0.01. ALT, alanine aminotransferase; AST, aspartate aminotransferase, ALP, Alkaline Phosphatase.

**Table 6:** Economic performance indicators for lambs fed experimental diets.

Parameter	Commercial Concentrate	Formulated Concentrate	*p*-Value
Concentrate Cost (OMR)	5.87 ± 0.96	2.05 ± 0.22	<0.001 ***
Rhodes grass Cost (OMR)	0.78 ± 0.16	0.71 ± 0.16	0.391
Total Feed Cost (OMR)	6.64 ± 1.01	2.77 ± 0.33	<0.001 ***
Cost per BWG kg (OMR/kg)	1.04 ± 0.30	0.38 ± 0.07	<0.001 ***
Economic Conversion Ratio	1.14 ± 0.33	0.41 ± 0.08	<0.001 ***
Cost Reduction (%)	—	58.3%	—
Cost Savings per Animal (OMR)	—	3.87	—

Values are presented as the mean ± SD. Statistical comparisons were performed using independent *t*-tests. *** *p* < 0.001. OMR = Omani Riyal (1 OMR ≈ 2.60 USD).

## Data Availability

The data presented in this study are available on request from the corresponding author.
